# Foreign Bodies in the Nose

**Published:** 1889-02

**Authors:** Frank Trester Smith

**Affiliations:** Chattanooga, Tenn., Late Assistant Surgeon New York Ophthalmic and Aural Institute


### FOREIGN BODIES IN THE NOSE.*

BY FRANK TRESTER SMITH, A. M., M. D., CHATTANOOGA, TENN., LATE ASSISTANT SURGEON NEW YORK OPHTHALMIC AND AURAL INSTITUTE.

Cases of foreign bodies in the nose are not rare, but the following present some features that it is believed render them worthy of your consideration:

*Case 1.*—Piece of hickory nut-shell in the nose seven months. Samuel Goldberg, *æt.* five. January 12th, 1887, occurred in my service at the Eastern Dispensary, New York City.

*History.*—Offensive breath, and discharge confined to the right nostril, lasting seven months. Discharge was at times bloody. Inspection showed a black mass, which to the probe gave the feeling of dead bone. It was situated some little distance from the tip of the nose, just below the middle turbinated bone. It was removed with little trouble with a pair of anatomical forceps.

* Read before the Tennessee State Medical Society.

After washing away the mucus, it proved to be a piece of hickory-nut shell of irregular shape, half an inch long, a quarter of an inch wide, and three-sixteenths of an inch in thickness. The operation was painful, as no anæsthetic was used. The child had been treated at several dispensaries, the mother stated, without relief, as the nature of the trouble was not discovered. No history of the entrance of the foreign body. Its presence was unknown until it was removed.

*Case 2.*—Glass button in the nose three years. Beginning formation of a rhinolith. Henrietta Kurz, *æt.* eight, 219 East 78th St.,New York, December 12th, 1886.

While examining the nose incidentally, during an examination for an eye affection, a foreign body was detected in the right nostril, situated anteriorly and between the middle and inferior turbinated bones. It was removed anteriorly with a pair of light mouse-tooth forceps, such as are used to steady the eye-ball during operations on that organ.

No anæsthetic was used. The operation produced a little bleeding and very little pain. The offending substance proved to be a button, which the mother recognized by some peculiarity as having belonged to a dress that had been worn out three years before. The button had been eaten away by the secretions, and afterwards had become incrusted with material, the beginning of rhinolith.

It is a little over three-eighths of an inch in diameter, with an eyelet projecting from one side. The history was that of nasal trouble of some considerable duration. The mother could not state the exact time, but thought two or three years. The trouble was confined to the right nostril. A one-sided discharge, with slightly offensive odor, being the principal symptom. For this the patient had been treated at the Mt. Sinai Hospital dispensary,and at another dispensary in the throat department, without relief. The right nostril would bleed sometimes quite freely. No history could be obtained of the entrance of the button. Neither the patient nor her mother were aware of its presence, but there was no doubt but that it had entered three years previously.

*Case 3.*—Rhinolith formed around a cherry pit in the nose thirteen years. Jacob Vogel, *æt.* twenty-one. January 26th, 1887. When eight years old, was taken by his father to consult a doctor in Germany for a discharge from the nose, which was confined to the right side. The discharge had continued more or less ever since, except that it would cease a short time every summer. It was more or less bloody. For the past two weeks there had been considerable itching on the right side. With this history, the patient came to me at the Hospital for Ruptured and Crippled, in New York. Inspection showed a swelling in the septum of the right nostril, and an irregular mass covered with mucus joining it.

To the probe it gave the feeling of dead bone. Clearing away the mucus, the foreign body could be plainly seen an inch from the entrance of the nostril. It was somewhat movable, being readily rotated on an axis at right angle to the septum. It was partly imbedded in the septum on the one side, and on the other in the inferior turbinated bone, which was considerably swollen. A twenty per cent. solution of cocaine was used for the two-fold purpose of reducing the swelling and producing anæsthesia. It was first attempted to extract with the delicate mouse-tooth forceps, but considerable pain was produced, and the object could not be moved. More cocaine was used to further reduce the swelling. The body was then seized with a strong pair of dressing forceps, and removed with considerable force and very little pain. It appeared to be a piece of bone from its irregular surface and peculiar appearance. It was about five-eighths of an inch long by a quarter wide and a quarter thick, and presented many irregularities. Examining it a month or so later, it was found to be a rhinolith having a cherry pit nucleus. This was incrusted with a formation of stony hardness. The cherry pit formed about half the mass. The patient was not aware of its presence, and could give no history of its entrance into the nose, where it had doubtless been for thirteen years.

In looking over the histories of these three cases, one is impressed with the presence of one symptom to which, so far as I know, no one has as yet called attention. I refer to the existence of a chronic discharge, confined to one nostril. It may be said that this is pathognomic of a rhinitis due to a foreign body. In all other forms both sides are affected. Should the discharge be sanious, the diagnosis is the more certain. Another point is that no history of the entrance of the body is given in any of the cases. This brings up the question as to how the entrance is effected. From the well-known habit that many children have of thrusting substances into the various cavities of the body, it is not improbable that they were thus introduced during play. The fact that they were all found in the right nostril might favor this idea. But the possibility of their introduction through the posterior nares must not be forgotten. Paulet relates a case where a patient could not eat peas without blowing one through his nose. Lowndes gives one where a metallic ring was found in the naso-pharynx, too large to have gone through the nostril. Cases are on record where, while drinking water, leeches have entered and attached themselves to the pharynx, and then found their way into the nasal cavity.

Hickman relates that a young girl, aged sixteen, had a brass ring in the naso-pharynx, which he removed. The ring was such as are used for purse slides, one inch in diameter and a quarter of an inch high. The mother related that, when the child was about two years old, she was one day seized with a sudden attack of suffocation, during which she carried her hand to her throat. The mother introduced her finger and felt the ring distinctly, but it was at once displaced and lost. She was supposed to have swallowed it. She had a discharge, at first sanious, and later muco-purulent, which had continued ever since. Recovery complete, after removal of the ring. The entrance of material into the nasal cavity, during emesis, is well known, and needs but to be alluded to at this place. During the act of swallowing, some sudden movement may disturb the regular play of the muscles and cause an expiratory effort, which may force particles of food past the soft palate. In paralysis of certain muscles, during deglutition, foreign bodies may find their way into the nasal cavity. Rarely they get into the Eustachian tube, the beard of barley has been found here post-mortem. A piece of whalebone was lost in the tube during catheterization, but was afterward extracted by the patient. More rarely are foreign bodies found in the frontal sinuses. The history is not always satisfactory, as in the cases related. Toleration is never established. Some very remarkable cases of this nature are on record. Among them is one related by Hayes, where a glass-button remained in the nasal cavity twenty years. Mackenzie cites a case where a piece of shell was retained seventeen years, and another where a musket-ball was not removed until after twenty-five years. Noyes removed the entire breech-pin of a gun from the nasal cavity. Its presence was due to an explosion. The patient consulted the doctor for his eyes. Verneuil found a rhinolith so large that it had to be crushed before extraction. Brown extracted one, one and three-quarter inches long, one inch wide, and half an inch thick. All kinds of particles have been found in the nose, buttons, beads, nuts, stones, peas, beans, various articles of iron, wood, glass, stone. Among animate objects, we have insects, leeches, worms.

The changes the substances undergo, seem to be about as follows: Most animate objects are killed, but it must be noted that leeches may attach themselves and live indefinitely. Lumbricoid worms may find their way from the intestines and remain a long time without being dislodged. Articles of iron are soon covered with a layer of oxide. Lead is covered with the sulphide. Glass first undergoes a corroding process. This is the same as that seen in an artificial eye, after it has been in use for perhaps a year, when it becomes so rough that it can no longer be worn without producing irritation. With the specimen presented, is another button, which shows its original size. This was given me by the mother of the little patient. The eyelet would indicate that the button was larger than it is at present.

It will be noticed further, that the button was covered with a very thin coating, which is the beginning formation of a rhinolith. Vegetable substances, if hygrometric, soon swell from the imbibition of moisture. Peas and beans have been known to sprout. Hard substances, such as the hickorynut shell, and the cherry pit, in the cases related, undergo no change in themselves, but seem to be more liable than other substances to form nuclei for rhinoliths. Any substance may form a nucleus for a rhinolith. The formation of these is well illustrated in the cases related. The nut-shell, being in situ only seven months, underwent no change. It was covered with a layer of altered secretion that was easily removed. The glass button, being retained three years, has a very slight incrustation, in some places not thicker than paper. The cherry pit was the nucleus of a rhinolith. The incrustation completely surrounded it, and was of such a nature as to appear like bone. Only when it was examined most carefully, a month or so later, was the error discovered.

These rhinoliths vary in composition. Generally, it may be said, they are formed from the sediment of the saline portion of the serum, phosphates of calcium and magnesium, choride of sodium, carbonates of sodium, calcium and magnesium, also the oxysulphate of calcium have been found bound together with animal matter. The important fact to be remembered is that they are friable. They can be crushed with little difficulty. It must also be remembered that they may be multiple. The formation of these calculi being similar to that of concretions in the bladder, their study is of double interest.

The symptoms of foreign bodies in the nose are such as we would expect *a priori*. The one which should be insisted upon is a one-sided rhinitis, and especially if the discharge is at times bloody. Primarily we have pain, a tendency to sneeze, epistaxis. If in the posterior portion, we have interference with deglutition, the timbre of the voice may be altered. Loss of smell, on one side, is not generally noticed, being masked by other symptoms.

The diagnosis depends on the care in investigating the nasal cavity. Most foreign bodies give the feel of dead bone. We may not be able in every case to distinguish from caries, but repeated examinations could hardly fail to clear up the diagnosis. Again caries being generally due to syphilis, there would be other signs of this disease. Calcarous degeneration of the mucus membrane of the nose is so very rare, that its consideration may almost be left out of the question. It should be remembered that rhinoliths may form beneath the mucous membrane.

*Treatment.*—Many expedients have been resorted to for the removal of offending substances. Sternutatories, to produce sneezing, are applicable in some cases. Emetics have been used, at the time of emesis the mouth being held closed, forcing the contents of the stomach through the nose. The method seems unnecessarily harsh. The use of the douche and syringe is mentioned only to be condemned. Danger to the middle ear is not simply theoretical. Insufflation has been practiced by two methods, first by blowing into the opposite nostril. It should not be forgotten that this may force matter into the Eustachian tube. Another method is to blow forcibly and suddenly into the patient's mouth. This seems more rational and is highly spoken of by those who have practiced it. It has the advantage of being easily applied even by laymen. The application of glue on a brush and then using traction has been recommended. Cohen uses a curved bougie introduced from behind. Gross recommends his ear-pick. Makenzie uses the douche. Bryan condemns syringes and resorts to the hook or the wire noose. Leeches may be loosened by using a solution of salt. It is well to bear all these various procedures in mind in dealing with a given case. Erichsen recommends the employment of anæsthetics in every case, But this is entirely unnecessary, especially since the introduction of cocaine.

There is a double indication for the use of the latter ; first to produce anæsthesia, second to reduce the swelling. This is accomplished by its constringing effect on the blood vessels, there being so much cavernous tissue it will be found that under the influence of cocaine the nostril will be much more patent. There is this precaution to be observed, *i. e.* to use less than three grains for fear of a toxic effect, that amount having caused death. After the swollen tissue has subsided the mouse-tooth or anatomical forceps may be tried, should these not succeed, stronger dressing forceps should be used. It must be born in mind that many foreign bodies may be crushed *in situ* and extracted piecemeal. This would spoil the specimen, however, and rob the doctor. Should we not succeed in getting the foreign body out anteriorly, it may be pushed through the posterior nares through into the pharynx thence out of the mouth. The danger of its falling into the larynx is not great, unless the patient is under the influence of an anæsthetic. To recapitulate then, the presence of a chronic discharge from the nose, especially if fœted should lead to a very careful examination. A one-sided discharge, particularly if bloody, is pathognomonic of the presence of a foreign body. In its removal the use of a very strong solution of cocaine, twenty per cent., is to be preferred to general anæsthesia. It should also be used to render the nose more patent. Thus it will assist in clearing up the diagnosis and in removing the foreign body. Weaker solutions than twenty per cent. require a much longer time to reduce the swollen tissue. Less than three grains of cocaine should be used as it is readily absorbed by the nasal mucous membrane. When thoroughly under the influence of cocaine, forceps are the most eligible means of removing anteriorly. Should it be so situated as not be easily extracted through the nostril, it may be removed through the posterior nares.

